# Indeterminate form of Chagas disease: historical, conceptual, clinical, and prognostic aspects

**DOI:** 10.1590/0037-8682-0254-2021

**Published:** 2021-07-23

**Authors:** Alejandro Marcel Hasslocher-Moreno, Sergio Salles Xavier, Roberto Magalhães Saraiva, Andréa Silvestre de Sousa

**Affiliations:** 1 Fundação Oswaldo Cruz, Instituto Nacional de Infectologia Evandro Chagas, Rio de Janeiro, RJ, Brasil.; 2 Universidade Federal do Rio de Janeiro, Faculdade de Medicina, Rio de Janeiro, RJ, Brasil.

**Keywords:** Indeterminate Form, Concept, Clinic, Prognostic, Chagas Disease

## Abstract

Chagas disease (CD) remains a serious endemic disease in Latin America and a major public health problem. Because of globalization, the disease has spread to non-endemic areas in the northern hemisphere. In the chronic phase of the disease, most patients present with the indeterminate form (IF), characterized by positive serology for *Trypanosoma cruzi*, absence of clinical findings, and normal findings in electrocardiogram (ECG). IF was not recognized as a clinical entity until decades after the discovery of the disease, and only in the 1940-50s, it was categorized as a form of CD, and its conceptual definition was ratified in the 1980s. Children, adolescents, and young adults with the IF benefit from etiological treatment and tend to have less progression to heart disease in the long term than the untreated ones. IF patients have an essentially benign clinical condition, and their prognosis can be compared to that of healthy individuals with normal ECG findings. Currently, because of aging, patients with the IF have comorbidities that require attention in health services.

## INTRODUCTION

Chagas disease (CD) remains a serious public health problem in Latin America^1^. Because of globalization, many immigrants with the disease, who are often asymptomatic, have moved to non-endemic areas in the northern hemisphere; this represents a new challenge in the fight against CD^2^ ^,^ ^3^. The natural history of CD has two phases: acute and chronic. The onset of the acute phase is near the moment of contamination. By contrast, the chronic phase occurs after the regression of the acute phase and has four well-defined clinical forms: 1) the indeterminate form (IF), which begins soon after the end of the acute phase and may persist throughout the patient’s life, during which the individual does not show symptoms and signs of CD and has normal findings in examinations of the heart (electrocardiogram) and digestive system (esophagography and opaque enema); 2) the cardiac form (CHD), which frequently involves rhythm and/or conduction heart disorders, left ventricular systolic dysfunction with or without heart failure, and thromboembolic phenomena; 3) the digestive form, which involves peristalsis dysfunction of the esophagus and/or intestine, and megasyndrome presentations; and 4) the mixed form, when cardiac and digestive manifestations occur simultaneously^4^.

IF is characterized by a latent period, and approximately half of the patients remain indefinitely^5^. It is defined as a disease stage wherein clinical evaluation, chest radiography, electrocardiogram (ECG), and contrast-enhanced esophageal and colon examination findings are normal^6^. Additional investigations using more sophisticated and sensitive complementary methods such as echocardiography (ECHO), cardiopulmonary stress test, 24-hour Holter, noninvasive autonomic tests, myocardial scintigraphy, and magnetic resonance imaging of the heart may show changes in a substantial number of the affected patients, which do not invalidate the current concept of IF^7^. 

The IF anatomical and histopathological substrate is represented by focal microscopic inflammatory lesions, in which parasites are rarely found using common histological methods^8^. The meaning of the lesions has been discussed. Some authors reported that the lesions would be cumulative and, with progression of time, would result in diffuse and confluent involvement of the myocardium^9^. By contrast, other authors report that the lesions would represent a state of parasite-host balance, without evolutionary potential^10^. In line with the latter hypothesis, focal myocarditis lesions in the IF were interpreted as being subject to a self-limited evolutionary cycle, balanced by the onset of some lesions and the discontinuation of others, which would allow a long host survival. In addition, it is postulated that the genetic factors of the patient are related to the diversity in the clinical course of CD^11^. Biomarkers for CHD progression are being studied in the immunological and molecular fields, in which enzymatic, humoral, and immunological interactions may be related to a set of genetic markers (polymorphisms) already existing, when the patient is infected, and these different responses to the parasite may determine disease progression^12^. 

IF generally has a good prognosis^6^ ^,^ ^13^ and it is quite similar to that in the general population with normal ECG findings^14^. In addition, IF is one of the main indications for the etiologic treatment of the disease^15^ ^,^ ^16^, which decreases the risk of vertical transmission^17^ and positively affects the costs of CD treatment^18^. 

## HISTORY AND CONCEPT

Carlos Chagas described the first case of American trypanosomiasis in 1909, and a clinical report indicated an acute febrile presentation^19^. At that time, the intrinsically progressive characteristic in the long term was not yet known. CHD was identified by Carlos Chagas in 1922^20^, and the IF of the disease was identified only years later. Curiously, the first case described in the literature (in a child called Berenice) was configured over the years as IF, and the patient died at 72 years of age without signs of heart disease^21^. The studies published in the first decades indicated that the IF was a transitory stage, wherein all individuals would have the potential to progress to CHD^22^.

The structuring of the Bambuí center^23^ led to the start of a new phase of CD clinical research, allowing the observation of the natural history of the disease^24^. From these studies, the IF was identified, and the concept of a mandatory transition stage began to be questioned. There was a long period between the first CD case reported and the first consensus on IF. In 1985, CD specialists at a scientific meeting in the city of Araxá-MG^25^ formally ratified the IF concept, i.e., as being characterized by positive serological and/or parasitological tests, absence of disease symptoms and/or signs, normal conventional ECG findings, and normal radiological findings of the heart, esophagus, and colon. After formalizing this definition, studies on the chronic form of CD, which were previously restricted to the approach of heart disease^22^, began to focus on IF^26^.

The word “indeterminate” was used for the first time by Carlos Chagas, and among the various names attributed to IF since then, the most widely used, although incorrectly, was “asymptomatic.” In the scientific and academic context, studies on patients with IF published in recent decades often refer to them as being asymptomatic. The use of this term is inappropriate, as patients with CHD may also have no symptoms of the disease. This led to the inappropriate use of the term "asymptomatic" in IF and CHD patients as if they had the same clinical condition, with important repercussions regarding the therapeutic and prognostic approach. 

Regarding another view, in 2010, the Scientific Committee on Chagas Disease of the Argentinean and Inter-American Federation of Cardiology^27^ criticized the term “indeterminate form” for understanding that this term implies a semantic interpretation, translating a sense of imprecise or uncertain and generating discomfort for doctors and patients in the use of this terminology in clinical practice. Based on this analysis, it was proposed that the term “indeterminate form” has changed to “without apparent pathology”.

Since the “Araxá meeting”^25^, there has been virtually no revision of the IF concept. A single proposition was made in 2002, when it was suggested for consideration, in addition to the absence of symptoms and changes to the electrocardiogram and radiological studies of the chest and esophagus, the two-dimensional echocardiographic demonstration of normality and the need for radiological demonstration of the colon, by opaque enema, in individuals without complaints of constipation should be abolished^28^. 

Cross-sectional studies conducted in endemic areas during 1973-1984 showed that, depending on the patient’s age, the prevalence of IF ranged from 63% to 100% in children, 39% to 58% in young adults, and stabilized at approximately 30% in people over 50 years of age^29^. The first longitudinal studies, all conducted in the field, reported the first information on IF's progression to the cardiac form. These studies intended to identify the rates of progression, using ECG as the monitoring method. These few prospective studies differed greatly in terms of patient age, number of cases, follow-up time, geographic area (latitude and longitude), permanence in the endemic area with active or controlled vector transmission, and migration to urban areas^13^. Unlike the studies published in the 1970s-1980s, the most recent longitudinal studies on the progression of IF have been carried out in urban areas, especially large cities with a population that is older and has been away from an endemic area for many years. A recent systematic review and meta-analysis of the progression of IF to the cardiac form showed progression rates ranging from 0.2% to 10.3%, with an estimated annual average of 1.9%^30^. 

## CLINICAL AND PROGNOSTIC ASPECTS

Patients with IF do not present with symptoms or signs related to CD. According to the classic definition, findings of the ECG and radiologic contrast examinations of the esophagus and colon are normal. Owing to limited access to the esophagus and, especially, colon in examinations, most patients are no longer evaluated using esophagography and/or opaque enema, which are tests considered the gold standard in the diagnosis of mega syndromes^28^. In addition, invasive and uncomfortable tests, such as colonoscopy, provide little practical and useful information for the clinical management of asymptomatic patients. Moreover, the tests involve ethical questions regarding beneficence and non-maleficence, as they are frequently considered bothersome and invasive examinations. Thus, in practical terms, patients with CD, normal ECG findings, and without digestive symptoms have been classified as "those without apparent cardiopathy" or even as those having IF, even without having undergone a complementary evaluation of the digestive system. Patients with IF are not always discernible from healthy controls, based on some or several functional and/or structural abnormalities detected in specific cardiac examinations^28^. Until recently, studies reported that there was no evidence that any of these cardiac abnormalities, either functionally and/or structurally detected in patients with IF, influenced the natural progression of the disease^28^. Currently, there are studies demonstrating that echocardiographic changes together with normal ECG findings have prognostic implications in IF^31^ ^,^ ^32^. Therefore, in a situation in which the patient has normal ECG findings and changes in the echocardiogram, the prognosis of this patient, in particular, is the same as that in another patient with an altered ECG and the same pattern of echocardiographic alteration.

Another aspect to be considered is that the presence of nonspecific changes in the ECG does not mischaracterize IF. Therefore, the isolated presence of electrical axis deviation to the left; low voltage, secondary ventricular repolarization change; sinus bradycardia ≥40 beats/min; sinus tachycardia; left anterior hemiblock; first-degree right or left branch block; first-degree atrioventricular block; single ventricular extrasystole; and migratory pacemaker in the ECG may not necessarily be related to CHD^33^.

Many factors have been considered to be involved in the risk of CHD development, including exposure to *T. cruzi* reinfection in areas with sustained vector transmission, male sex, parasite load, genetic aspects of the host, nutritional status, presence of comorbidities, and the social context and quality of life of CD patients^13^ ^,^ ^34^ ^-^ ^39^. A favorable medium-term prognosis for IF patients was demonstrated in longitudinal studies^6^, confirming that the mortality rates are similar between IF patients and people without the disease in the same age range^14^. Likewise, the annual incidence of sudden death in CD patients with normal ECG findings is low and similar to that in the population without CD^40^. However, some authors state that IF may involve a high risk of sudden death, although no study has been specifically designed to explain this issue^41^. In addition, patients with normal ECG but who have ventricular extrasystole on Holter or ergometry should be monitored and advised regarding the activity of daily living that requires intense or continued effort.

Regarding the etiological treatment in the chronic phase of the disease, national and international guidelines have considered IF as the main indication, particularly in children, adolescents, and young adults^15^ ^,^ ^16^. The earlier the treatment, the better is the effectiveness^6^. Despite the good prognosis, an annual rate of clinical progression of 1.9% justifies the etiological treatment to prevent CD progression^42^ and the incidence of events related to morbidity and mortality^43^.

IF became a worrying medical-social problem in the 1960s and the 1970s^44^, as many patients with this clinical form were considered incapacitated for work in labor selection tests^29^. These patients should not be indiscriminately excluded from activities that require habitual or heavy physical effort. However, as individual behavior is quite variable, every patient deserves to be assessed individually and in accordance with the intended work activity. There is still a need to emphasize the good prognosis of IF, which will prevent stigmatization and discrimination in the labor market. 

In the next few years, all indicators would show that health professionals will have to deal predominantly with patients infected a few decades ago and present mostly IF^8^. This situation is associated with the mean age of these patients, most of whom are older adults^45^. Although this group of older patients has a low potential for disease progression, it is subject to cardiac changes frequently associated with other cardiovascular diseases such as systemic arterial hypertension and coronary artery disease^46^. Furthermore, the presence of comorbidities such as diabetes mellitus, dyslipidemia, obesity, and depression requires proper clinical management^47^ ^,^ ^48^.

## CONCLUSION

From the evidence available to date, IF is associated with an essentially benign clinical condition. Consequently, these patients can generally manage everyday life without physical or labor restrictions. They should be informed of their usually favorable prognosis, offering etiological treatment according to age, to avoid possible clinical progression to heart disease, and be reassured about their health status. Patients exercising functions of greater risk or requiring effort must be monitored periodically. It is essential to prevent and avoid the stigma that usually permeates the personal, family, social, and professional lives of these patients. Currently, patients with IF are usually older and have comorbidities that require care in health services.
